# Continuous Aspiration Thrombectomy in High- and Intermediate-High-Risk Pulmonary Embolism in Real-World Clinical Practice

**DOI:** 10.1155/2020/4191079

**Published:** 2020-08-21

**Authors:** Aleksander Araszkiewicz, Sylwia Sławek-Szmyt, Stanisław Jankiewicz, Bartosz Żabicki, Marek Grygier, Tatiana Mularek-Kubzdela, Zbigniew Krasiński, Maciej Lesiak

**Affiliations:** ^1^1^st^ Department of Cardiology, Poznan University of Medical Sciences, 61-848 Poznan, Poland; ^2^Department of Vascular Surgery, Collegium Medicum of Zielona Gora University, 65-417 Zielona Gora, Poland; ^3^Department of Vascular, Endovascular Surgery, Angiology and Phlebology, Poznan University of Medical Sciences, 61-848 Poznan, Poland

## Abstract

**Objectives:**

We sought to assess the technical and clinical feasibility of continuous aspiration catheter-directed mechanical thrombectomy (CDT) in patients with high- or intermediate-high-risk pulmonary embolism (PE).

**Methods and Results:**

Fourteen patients (eight women and six men; age range: 29–71 years) with high- or intermediate-high-risk PE and contraindications to or ineffective systemic thrombolysis were prospectively enrolled between October 2018 and February 2020. The Indigo Mechanical Thrombectomy System (Penumbra, Inc., Alameda, California) was used as CDT device. Low-dose local thrombolysis (alteplase, 3–12 mg) was additionally applied in three patients. Technical and procedural success was achieved in 14 patients (100%). Complete or nearly complete clearance of pulmonary arteries was achieved in nine patients (64.3%), whereas partial clearance was achieved in five (35.7%). A significant improvement in the pre- and postprocedural patients' clinical status was observed in the following fields (median; interquartile range): heart rate (110; 100–120/min vs. 85; 80–90/min; *p* < 0.0001), systolic blood pressure (106; 90–127 mmHg vs. 123; 110–133 mmHg; *p* = 0.049), arterial oxygen saturation (88.5; 84.2–93% vs. 95.0; 93.8–95%, *p* = 0.0051), pulmonary artery systolic pressure (55; 44–66 mmHg vs. 42; 34–53 mmHg; *p* = 0.0015), Miller index score (21.5; 20–23 vs. 9.5; 8–13; *p* < 0.0001) and right ventricular/left ventricular ratio (1.3; 1.3–1.5 vs. 1.0; 0.9–1.0; *p* < 0.0001). No major periprocedural bleeding was detected.

**Conclusions:**

CDT is a feasible and promising technique for management of high- or intermediate-high-risk PE to decrease thrombus burden, reduce right heart strain, and improve hemodynamic and clinical status. Some patients may benefit from simultaneous local low-dose thrombolytic therapy. Nevertheless, its criteria and role in CTD-managed patients require further elucidation.

## 1. Introduction

Pulmonary embolism (PE) is the third leading cause of cardiovascular mortality and its occurrence is estimated at 39–115 cases/100000 inhabitants/year [1–4]. The presentation of PE may vary from asymptomatic or mild exertion disturbances (low-risk PE) treated with anticoagulants only to hemodynamic “obstructive” collapse and death (high-risk or massive PE) [5]. Systemic thrombolysis (ST) decreases mortality and improves clinical status in high-risk PE patients, but it also poses higher major bleeding risk [6]. The controversy concerns especially risk stratification and treatment in intermediate-high-risk (submassive) PE patients. Patients in this group do not have developed features of hemodynamic instability (hypotension, the need for catecholamines, disturbances, or loss of consciousness), but they have right ventricular (RV) overload characteristics, elevated markers of necrosis, and high values of Pulmonary Embolism Severity Index (PESI). In patients with intermediate-high-risk PE, ST is not indicated because the potential benefits do not outweigh the risk of serious bleeding complications [6, 7]. On the other hand, a significant percentage of intermediate-high-risk patients with PE may experience rapid hemodynamic deterioration and then the prognosis in this group is significantly worse. According to the present European and American guidelines, in the case of contraindications to ST or its failure, surgical embolectomy (SE) is recommended [5, 8]. However, a severe preoperative condition and high incidence of comorbidities as well as limited availability of “on-time” cardiac surgery result in high mortality in these patients. Due to the above conditions, risk assessment and qualification for different treatment methods should be dealt with by the institutional multidisciplinary team set-up for the treatment of PE (Pulmonary Embolism Response Team, PERT) [9–11]. Consequently, current treatment modalities such as anticoagulation (AC), ST, or SE have recently been expanded to modern transcatheter techniques, such as catheter-directed thrombolysis or mechanical catheter-directed aspiration thrombectomy (CDT) [12, 13]. The issue of choosing the catheter-directed modality determined by the mortality risk and contraindications to ST is widely discussed. Catheter-directed thrombolysis has been applied as an alternative to ST and has been shown to improve RV strain. Nonetheless, it is still associated with a relatively high bleeding risk, because of usage of thrombolytic agents [14]. As a result, CDT without thrombolytic drug administration is considered to be a promising therapeutic option [12, 13]. The present study aimed to evaluate the safety and feasibility of this approach.

## 2. Materials and Methods

**Figure 1:**
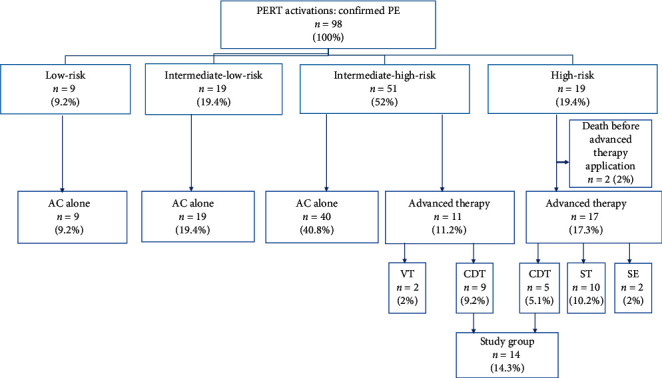
Detailed risk stratification of patients with PE and applied treatment modalities by our institutional PERT. AC = anticoagulation, advanced therapy = anticoagulation together with CDT or SE, ST, or VT, CDT = catheter-directed mechanical aspiration thrombectomy, PE = pulmonary embolism, PERT = pulmonary embolism response team, SE = surgical embolectomy, ST = systemic thrombolysis, and VT = venous thrombectomy.

Ninety-eight consecutive patients with acute PE were evaluated and screened by our institutional PERT between October 2018 and February 2020. Patients older than 18 years of age with proximal PE confirmed by computed tomography pulmonary angiography (CTPA) and symptoms onset within 14 days were eligible for enrollment in the present study. The severity of PE was categorized as low-risk, intermediate-low-risk, intermediate-high-risk, or high-risk in accordance with the guidelines of the European Society of Cardiology (ESC) [5, 15]. The precise PE patients risk stratification is presented on Figure 1. The details concerning organizing and functioning of our institutional PERT were published elsewhere [16]. In brief, PERT recommendation included AC therapy alone or advanced therapy defined as implementation of the AC together with some kind of invasive strategy including CDT or pharmacomechanical thrombectomy (CDT along with low dose of the thrombolysis with the alteplase), ST, SE, or venous thrombectomy (VT). All high-risk PE patients with absolute contraindications to ST or its failure (refractory circulatory collapse) who were not eligible for SE were qualified for CDT after careful assessment by PERT. Intermediate-high-risk patients with RV dysfunction confirmed by imaging (CTPA or transthoracic echocardiography) and elevated troponin concentration with concomitant: (1) systolic blood pressure (SBP) > 90 mmHg and ≤100 mmHg, (2) heart rate (HR) ≥ 110/min, or (3) arterial blood oxygen saturation (SaO_2_) <90% during spontaneous breathing (atm) were closely monitored on the cardiac intensive care unit. All patients were reevaluated after 24 hours of AC therapy. If at least one of the above factors persisted, the patient was qualified for CDT. In case of clinical deterioration (sudden occurrence of one or more of the above-mentioned factors), the patient was also qualified for CDT. Exclusion criteria were pregnancy, refusal to sign the informed consent form, presence of intracardiac thrombus, diagnosed thrombophilia or severe thrombocytopenia (platelet count below 20 000 *μ*L), history of severe or chronic pulmonary hypertension, serum creatinine concentration higher than 1.8 mg/dl, and known serious and uncontrolled sensitivity to radiographic agents (see also Supplementary material Table 1).

**Figure 2:**
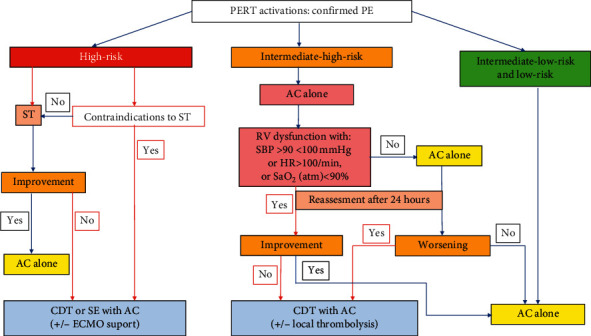
Schematic decision-making approach for PE management and qualification for CDT. AC = anticoagulation, CDT = catheter-directed mechanical aspiration thrombectomy, ECMO = extracorporeal membrane oxygenation, HR = heart rate, PE = pulmonary embolism, PERT = pulmonary embolism response team, RV = right ventricle, SaO_2_ = arterial oxygen saturation, SBP = systolic blood pressure, and ST = systemic thrombolysis.

Among the whole study population, 14 patients with high- or intermediate-high-risk PE were qualified to perform CDT. The detailed indications to CDT in particular cases were presented in Supplementary material Table 2. Patients with intermediate-low‐risk and low‐risk PE were monitored and treated with AC according to the ESC guidelines [5, 15]. The management algorithm is presented in Figure 2.

The study protocol of this prospective observational study was in accordance with the Declaration of Helsinki and was approved by the Institutional Ethics Committee (approval number 879/19). All the patients accepted the treatment and gave an informed consent to participate in the registry (if they were unconscious, family members approved the treatment).

### 2.1. Catheter-Directed Thrombectomy

**Figure 3:**
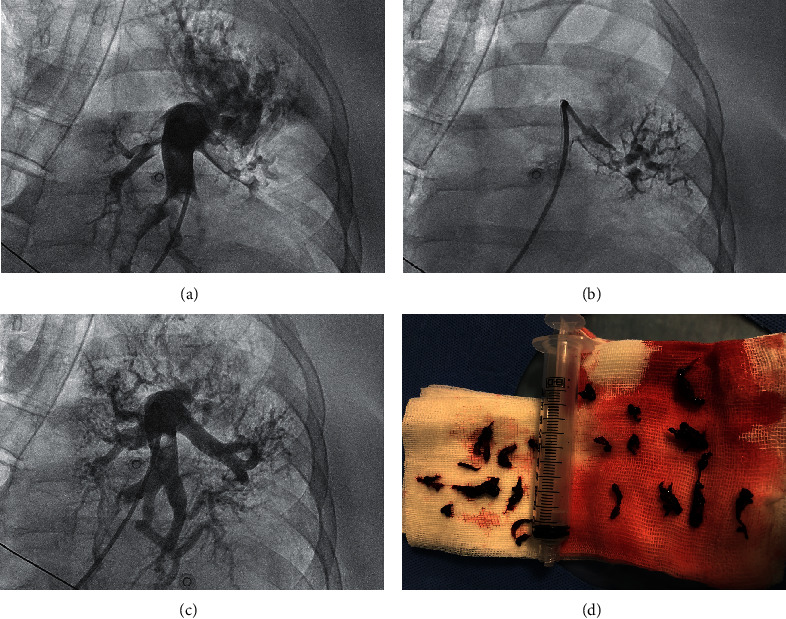
The CDT procedure. CDT = catheter-directed mechanical aspiration thrombectomy. (a) An initial angiogram illustrating the thrombus location in the left lobar arteries. (b) The Indigo catheter is placed in the target clots. (c) An angiogram after the CDT revealing marked improvement in the flow to the inferior lobe of the left lung. (d) An image of the removed clots.

Common femoral venous access was obtained by placing 6F vascular sheath. The intravenous bolus of 5000 IU of unfractionated heparin (UFH) was administered unless absolute contraindications were present. The main pulmonary artery was catheterized by using a pigtail diagnostic catheter, and an initial pulmonary angiogram was performed to demonstrate the location and extent of thrombi in pulmonary arteries (see Figure 3). Then, pulmonary arterial pressure (PAP) was measured according to the current guidelines [17]. Subsequently, a 115 cm, 8F CDT catheter (Indigo CAT8 XTORQ; Penumbra, Alameda, California) was advanced through a 90 cm, 8F Flexor sheath (Cook; Bloomington, Indiana) to perform procedure. A direct-aspiration first-pass technique was performed purposefully to attach a large thrombus to the catheter tip by suction and then pull it out through the sheath. If the first-pass technique was ineffective, a separator wire was repeatedly passed through the thrombus to break it up and simplify aspiration. Occasionally, a Judkins right coronary diagnostic catheter or multipurpose catheter was used to facilitate access to the lobar or segmental arteries and then were replaced by the Indigo CAT8 XTORQ catheter. The decision to terminate the intervention was at operator's discretion after careful evaluation of hemodynamic parameters (restoration of the SBP≥100 mmHg, HR < 100/min), improvement of SaO_2_ ≥ 92%, practicable clot burden reduction, and total amount of aspirated blood (should not exceed 300 ml) [18–20]. An additional low dose (1 mg per hour per patient through 6–12 hours) of the thrombolytic drug alteplase was administered to patients with an assessed angiographic clot burden reduction below <50%. If PE symptoms onset was within 12–14 day prior the procedure, a low dose (3 mg) of alteplase per patient was applied through a perforated angioplasty balloon as an introduction to CDT in order to better prepare the thrombus for aspiration (a hybrid strategy). All procedures were performed by two experienced interventional cardiologists (AA and SJ). After CDT, UFH or low molecular weight heparin (LMWH) in weight-adjusted dose were continued for 24–48 hours depending on clinical state, and then direct oral anticoagulant was introduced at physician's discretion.

### 2.2. Data Collection and Study Endpoints

The recorded data included demographics, personal medical history, clinical findings (presenting symptoms, hemodynamic and pulmonary status, along with echocardiographic results taken before and 24 hours after the procedure), and outcomes (including major procedure-related complications or bleeding events).

Technical success was defined as targeted placement of the devices, initiation of aspiration thrombectomy, and obtaining the embolic material. The clinical endpoints of the study were defined as follows: (1) clinical improvement during CDT procedure (change in SBP, HR and SaO_2_); (2) improvement of PAPs during the CDT procedure; (3) reduction in vascular obstruction in the angiography measured at the end of CDT with Miller Index score [21]; (4) change in right ventricle (RV) strain in echocardiography measured 24 hours after the procedure; (5) death caused by PE (RV failure) during index hospitalization or follow-up period; (6) death of any cause during index hospitalization or follow-up period; (7) CDT-related major adverse events (major bleeding, pulmonary vascular injury). See also Supplementary material Table 3.

To assess the extent of angiographic reduction in thrombus burden, pulmonary angiograms performed before and immediately after the procedure were reviewed by two independent observers. To quantify the degree of pulmonary arterial obstruction, the Miller index score was calculated as described previously [21]. The reduction of thrombus burden was classified as complete clearance (>90% reduction in Miller index score), nearly complete (50%–90% clearance), or partial (<50% clearance).

An independent cardiologist qualitatively assessed right heart strain with echocardiography, measuring right ventricular/left ventricular (RV/LV) ratio, tricuspid annular plane systolic excursion (TAPSE), RV and LV diameter, and systolic wave.

Bleeding events were classified according to Valve Academic Research Consortium-2 guidelines criteria published elsewhere [22]. Life-threatening (disabling) bleeding was defined as bleeding into critical organ or causing hypovolemic shock or drop in hemoglobin concentration more than 5 g/dL. Major bleeding was defined as bleeding causing drop in hemoglobin concentration more than 3 g/dL, but not fulfilling the criteria of life-threatening bleeding. Clinically significant bleedings which did not fullfill the criteria of life-threatening and major bleeding were categorized as minor bleedings (such as access site hematoma) [22]. The Charlson Comorbidity Index score was also calculated for each patient [23]. All survived patients were evaluated on out-patient follow-up visit one month and three months after CDT procedure.

### 2.3. Statistical Analysis

Patient characteristics are expressed as absolute and percentage frequencies for categorical variables and median and interquartile range (IQR) for continuous variables. The normality distribution was assessed with the Shapiro-Wilk test. Continuous variables were compared using depending Student's *t*-test or Wilcoxon signed-rank as appropriate. A two-tailed alpha of 0.05 was considered statistically significant. Statistical analysis was performed using Statistica 13.7 version (StatSoft, Inc., Tulsa, Oklahoma, USA).

## 3. Results

Among all PERT patients, 51 (52%) were categorized as intermediate-high-risk and 19 (19.4%) as high-risk PE (see Figure 1). Fourteen patients underwent CDT (8 female and 6 male). Among these patients, 5 high-risk PE patients were qualified for CDT on the basis of absolute contraindication to ST and refractory circulatory collapse, and 9 intermediate-high PE patients were qualified for the CDT procedure because of coexistence of persistent RV dysfunction with tachycardia HR ≥ 110/min, SBP<100 mmHg, and SaO_2_ < 90% (4 patients) or persistent RV dysfunction with concomitant tachycardia ≥ 110/min and SaO_2_ < 90% (5 patients). The median age of patients who underwent CDT was 56 years (range: 29–71 years) and median body mass index was 26.8 kg/m^2^ (IQR: 23.9–29.7 kg/m^2^). Baseline clinical, echocardiographic, and laboratory characteristics are summarized in Table 1.

Technical success was achieved in all 14 (100%) patients. The median duration of CDT procedure was 72 minutes (IQR: 60–100 minutes) and the median blood loss was 300 ml (IQR: 290–320 ml). Complete reduction in thrombus burden was achieved in 2 patients (14.3%), nearly complete in seven patients (50%) and partial in five (35.7%) patients. The pharmacomechanical treatment (CDT along with local thrombolysis) was implemented in three patients, including two patients with saddle thrombus and the clot clearance <50% as a supplement after CDT (these patients received a total dose of 6 mg of alteplase). In one patient with the longest duration of PE symptoms, a hybrid strategy was applied. The precise data are shown in Table 2. No patient died because of RV insufficiency. There was a significant clinical status improvement after CDT. The median HR significantly decreased from median 110/min to 85/min (*p* < 0.0001), while median SBP increased from 106 mmHg to 123 mmHg immediately after CDT (*p*=0.049). Blood SaO_2_ also significantly increased after CDT, from median 88.5% baseline to median 95% after CDT (*p*=0.0051).

The PAP significantly decreased immediately after the procedure. The median change in systolic PAP was 10.5 mmHg (IQR: 7–17 mmHg), while the median change in mean PAP was 6.5 mmHg (IQR: 5–8.5 mmHg), respectively. The median Miller index score before CDT was 21.5 (IQR: 20–23), while after CDT median Miller index score was 9.5 (IQR: 8–13)(*p* < 0.0001).

**Figure 4:**
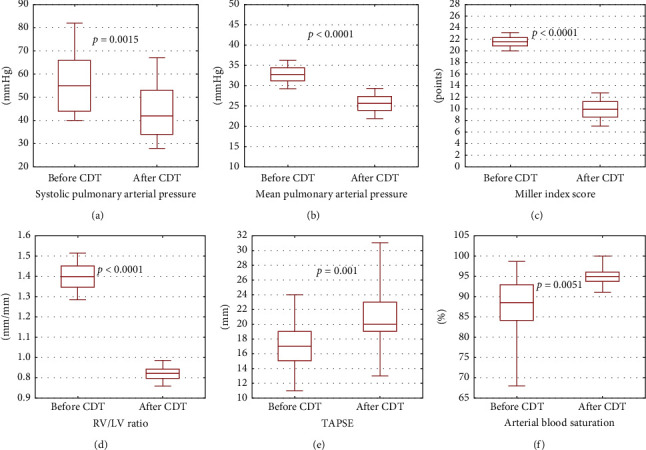
Hemodynamic and echocardiographic outcomes before and after CDT. CDT = catheter-directed mechanical aspiration thrombectomy, LV = left ventricle, RV = right ventricle, and TAPSE = tricuspid annular plane systolic excursion. (a) Change in systolic arterial pressure. (b) Change in mean pulmonary arterial pressure. (c) Change in Miller index score. (d) Change in right ventricular/left ventricular ratio. (e) Change in tricuspid annular plane systolic excursion. (f) Change in arterial blood saturation.

In all cases, RV/LV ratio (median 1.3 before CDT vs. median 1.0 after CDT, *p* < 0.0001) and TAPSE (median 16 mm before CDT vs. median 20 mm after CDT, *p*=0.001) significantly improved 24 hours after the procedure. The detailed data are presented in Table 3 and Figure 4.

One patient died (43-year-old female patient after >60 min; cardiopulmonary resuscitation before admission; CTD performed on extracorporeal circulation support) due to irreversible brain injury and pneumonia, two days after the procedure. No life-threating or major bleeding related to the CTD procedure was observed. We observed minor bleedings, limited inguinal hematoma in two (14.3%) patients after the procedure. No interventions were necessary in those patients. Two patients required red blood cells transfusion during hospitalization period (both of them had moderate anemia at baseline due to neoplastic disease or previous surgery which was unrelated to CDT).

## 4. Discussion

The results of our small observational study suggest that CDT might be an alternative or supplemental to standard management, especially in patients with contraindications to ST or SE. The CDT approach allows removing thrombi from the pulmonary arteries without the additional risks posed by thrombolysis or cardiac surgery [12, 13]. Early treatment restoring patency of occluded pulmonary arteries seems to be the crucial factor affecting mortality in high-risk PE. The efficient clot removal improves the patient's condition and alleviates the symptoms in intermediate-high-risk patients. Possibly, the reduction of thrombotic material burden may also decrease the probability of postembolic disease including chronic thromboembolic pulmonary hypertension. According to the current ESC guidelines, shock or systemic hypotension is an accepted indication for urgent thrombolysis in patients with acute PE and also the therapy of choice in case of deterioration in previously hemodynamically stable patients [5–7]. Surgical or endovascular thrombectomy can be an option in selected cases. The guidelines suggest also the endovascular approach as an alternative for SE in patients with unsuccessful ST or contraindication to thrombolytic therapy [5]. In intermediate-high-risk PE patients, ST may pose the bleeding risk that overweighs potential benefits [7]. On the other hand, the decision on thrombolysis or surgical therapy is often taken too late, and hemodynamic deterioration in this group of patients is associated with high mortality.

Recently, other percutaneous modalities have also garnered an interest because of their ability for restoration of pulmonary perfusion without significant increase in bleeding risk. These involve a variety of system including not only thrombus aspiration, but also mechanical fragmentation or mechanical or ultrasound-assisted thrombus fragmentation with local administration of a small dose of thrombolytics [24–29].

Despite the rapid development of transcatheter techniques, limited data are available on the efficacy and safety of this therapy, and experience is based on a small number of single-arm observational studies or case series. So far, only a few studies on the use of Indigo Penumbra CDT in PE patients have been published [18, 30–32]. Al-Hakim et al. used Indigo to treat six patients with submassive PE and a contraindication to thrombolysis [31]. They found significant reduction in systolic PAP, RV/LV ratio, Miller index, and CT obstructive index. There were no procedural or periprocedural complications. Pieraccini et al. treated 18 patients with massive (8/18) or submassive (10/18) PE [32]. Technical and procedural success was achieved in all patients; however, four patients died during index hospitalization (mostly because of serious comorbidities). Nevertheless, the authors showed a significant improvement in the pre- and postprocedural RV/LV ratio, pulmonary SaO_2_, heart rate, PAP, and Miller index score [32]. In another similar study, Ciampi-Dopazo et al. presented the results of treatment in 18 patients with PE [18]. The procedure was considered a technical success in 17 patients (94.4%) and a clinical success in 15 (83.3%); three patients died. Two patients died of massive PE, and one died due to submassive PE. Echocardiography showed significant improvements in RV size, TAPSE, and systolic wave. Two patients who received previous systemic fibrinolytic agents experienced intracranial bleeding, and abdominal bleeding developed in one patient [18]. In our series, we achieved also high rate of the technical and clinical success. All the patients who survived had fast and significant clinical improvement. We have achieved improvements in RV strain markers, significant reduction in PAPs, and improved echocardiographic and even electrocardiographic markers as we reported previously [33].

Mechanical thrombectomy with the Indigo system has several important limitations. The first one is the risk of peripheral embolization. During the procedure, it is possible to shift embolic material from large main or lobar pulmonary arteries to the numerous segmental arteries resulting in an increase in pulmonary vascular resistance and rapid clinical deterioration instead of improvement. One should take care of axial position of the catheter and continue the procedure in distal arteries until the flow is restored [33]. The next problem is blood loss following the extensive and long-lasting procedure. In our center, we stopped the treatment if the loss exceeded 300–350 ml. Another limitation of thrombectomy with the Indigo system is the difficulty in removing a large and long thrombus originating, for example, from the iliac vein. The catheter diameter (8F) is usually too small in this case. Although a large-bore device (FlowTriever) that mechanically engages thrombus was lastly proposed for such clinical scenarios, the system in not currently available outside the United States [26]. In our group, we used pharmacomechanical treatment in three patients (in two patients as a supplement after CDT, and in one patient as an introduction to CDT in order to facilitate thrombus for aspiration). Despite the possibility of rheolytic thrombectomy for the large clot removal with AngioJet catheter, it should not be used in initial PE treatment because of safety concerns [13].

In case of large thrombus fragmentation, partial thrombectomy should be performed, and in the absence of absolute contraindications, local low-dose thrombolytic therapy (e.g., alteplase 1 mg/*h* for 6–12 hours) might be also applied. In our group, we obtained no complete removal of thrombi in up to 80% of patients. However, we obtained partial or complete clearance in over 60%. This indicates that even partial success of thrombectomy can lead to rapid and effective hemodynamic and clinical improvement, as long as it is supplemented with anticoagulation, which remains the basis of PE treatment. Even partial improvement of pulmonary flow can restore sufficient cardiac output and reverse RV strain.

We found Indigo system especially effective in patients with thrombi in lobar and segmental arteries. These patients constitute the majority of our study group. In our opinion, saddle or central PE should be treated with SE unless bigger devices like FlowTriever are available [26].

Some previous studies reported a close correlation of early PE mortality with patients' age and comorbidities [34, 35]. The multimorbidity assessed by Charlson Comorbidity Index, which comprises among others myocardial infarction, chronic lung disease, malignancy, or renal failure, was found to be the most important prognostic factor of in-hospital death for patients with acute PE [35]. In present study the median Charlson Comorbidity Index was 2 (IQR: 1–3) which indicates that PE patients were moderately ill prior to PE occurrence. The most frequent concomitant diseases were obesity, arterial hypertension, and malignancy.

The present study is burdened by several limitations. One of the study limitations is its observational nature, which may also be considered as a strong point and reflects the real-life nature of the study. The study included a small number of patients from a single center. Despite the statistical significance observed in variables after intervention, the small number of studied patients limits the extrapolation of these results.

## 5. Conclusions

Continuous aspiration mechanical thrombectomy with Indigo system is a feasible and promising technique for management of high- and intermediate-high-risk PE to decrease thrombus burden, reduce right heart strain, and improve hemodynamic and clinical status. Some patients, such as those with a severe clinical course and a particularly large initial thrombus burden or those with a large residual clot burden, may benefit from an additional, local, low-dose thrombolytic therapy. Further studies are needed to define clinical and angiographic scenarios for the hybrid strategy.

## Figures and Tables

**Table 1:** Detailed clinical and outcomes characteristics.

Variables	*N* = 14
Male/female	6/8
Age (years)	56 (44–65)
Body mass index (kg/m^2^)	26.8 (23.9–29.7)
Comorbidities	
Arterial hypertension	5 (35.7%)
Diabetes mellitus	2 (14.3%)
Obesity	8 (57.1%)
Coronary artery disease	0 (0%)
Chronic obstructive pulmonary disease	1 (7.1%)
Charlson Comorbidity Index	2 (1–3)
Smoking	2 (14.3%)
Risk factors of PE	
Contraceptives	0 (0%)
Immobilization/surgery during last 14 days	8 (57.1%)
Malignancy	4 (28.6%)
Prior PE or DVT	0 (0%)
Clinical presentation	
Shock	3 (21.4%)
Syncope or presyncope	5 (35.7%)
Cardiac arrest	2 (14.3%)
Respiratory failure (desaturation <92%)	8 (57.1%)
Dyspnea	12 (85.7%)
Chest pain	1 (7.1%)
DVT at presentation	9 (64.2%)
HR (per min)	110 (100–120)
Respiratory rate (per min)	32.5 (30–34)
SBP (mmHg)	106 (90–127)
DBP (mmHg)	73.5 (59–87.5)
Laboratory findings	
Troponin I positive at presentation	14 (100%)
Troponin I concentration (ng/ml)	59.4 (0.29–64.5)
D-dimers concentration (ng/ml)	11900 (4244–17185)
NT-proBNP concentration (pg/ml)	3993.5 (2457–47670)
Echocardiographic findings (4CH)	
RV diameter (mm)	46 (44–46)
LV diameter (mm)	34 (32–26)
RV/LV ratio (mm)	1.3 (1.3–1.5)
TAPSE (mm)	16 (13–19)
LV ejection fraction	55 (50–55)
RV systolic pressure	60 (42–62)
In-hospital death	1 (7.1%)
Death of RV insufficiency	0 (0%)
Death during follow-up	2 (14.3%)
Minor bleeding	2 (14.3%)
Major bleeding	0

Values are median (IQR) or *n* (%); 4CH = four chamber view, DBP = diastolic blood pressure, DVT = deep vein thrombosis, HR = heart rate, PE = pulmonary embolism, NT-proBNP = N-terminal pro-B-type natriuretic peptide, LV = left ventricle, RV = right ventricle, SBP = systolic blood pressure, and TAPSE = tricuspid annular plane systolic excursion.

**Table 2:** The CDT procedure characteristics.

Variables	*N* = 14 (100%)
PE location	
Bilateral	14 (100)
Saddle and main arteries	2 (14.3)
Lobar and segmental	9 (64.3)
Segmental and subsegmental	3 (21.4)
Clot clearance	
>90%	2 (14.3)
50–90%	8 (57.1)
<50%	4 (28.6)
Access site	
Femoral	13 (92.9)
Jugular	1 (7.1)
Time from PE diagnosis to CDT (days)	2.5 (1–13.5)
CDT duration time (min)	72 (60–100)
Radiation dose (mGy)	159 (113–273)
Fluoroscopy time (min)	24.5 (20–36)
Contrast use (ml)	207 (150–275)
Amount of blood loss (ml)	300 (290–325)
AC type before procedure	
UFH	9 (64.3)
LMWH	5 (35.7)
Length of hospitalization (days)	6 (4–7)

Values are *n* (%) or median (IQR). CDT = catheter-directed mechanical aspiration thrombectomy, UFH = unfractionated heparin, and LMWH = low molecular weight heparin.

**Table 3:** Comparison of hemodynamic and echocardiographic outcomes before and after CDT.

Variables	Before CDT	24 hours after CDT	*p*
Systolic pulmonary arterial pressure (mmHg)	55 (44–66)	42 (34–53)	0.0015
Mean pulmonary arterial pressure (mmHg)	32 (29–37)	26 (20–30)	<0.0001
Diastolic pulmonary arterial pressure (mmHg)	20 (17–23)	14 (12–17)	0.0006
Miller index score	21.5 (20–23)	9.5 (8–13)	<0.0001
RV/LV ratio	1.3 (1.3–1.5)	1.0 (0.9–1.0)	<0.0001
TAPSE (mm)	16 (13–19)	20 (19–23)	0.001
Arterial blood saturation (%)	88.5 (84.2–93)	95 (93.8–96)	0.0051
HR (per minute)	110 (100–120)	85 (80–90)	<0.0001
SBP (mmHg)	106 (90–127)	123 (110–133)	0.049

Values are median (IQR). CDT = catheter-directed mechanical aspiration thrombectomy, HR = heart rate, LV = left ventricle, RV = right ventricle, SBP = systolic blood pressure, and TAPSE = tricuspid annular plane systolic excursion.

## Data Availability

The data used to support the findings of this study are available from the corresponding author upon request.

## Supplementary Materials

Supplementary Materials Table 1: inclusion and exclusion criteria for CDT procedure. Table 2: clinical presentation of study patients, indications to CDT, and PE risk classification. Table 3: the clinical endpoints of the study.
