# Supplementary material for: Continuous Aspiration Thrombectomy in High- and Intermediate-High-Risk Pulmonary Embolism in Real-World Clinical Practice
Source: J Interv Cardiol. 2020 Aug 21;2020:4191079. doi: 10.1155/2020/4191079 (PMC7456496; doi:10.1155/2020/4191079)
Supplement: Supplementary Materials — Table 1: inclusion and exclusion criteria for CDT procedure. Table 2: clinical presentation of study patients, indications to CDT, and PE risk classification. Table 3: the clinical endpoints of the study. [file 4191079.f1.docx]

**Supplementary Material**

Table 1. Inclusion and exclusion criteria for CDT procedure.

CDT= catheter-directed mechanical aspiration thrombectomy, CTPA= computed tomography pulmonary angiography, HR= heart rate, PE=pulmonary embolism, RV= right ventricle, SaO_2_= arterial blood oxygen saturation, SBP= systolic blood pressure, SE=surgical embolectomy, ST= systemic thrombolysis

| Inclusion criteria | Exclusion criteria |
| --- | --- |
| 1. Clinical symptoms and presentation consistent with PE | 1. Pregnancy |
| 2. PE symptoms duration ≤ 14 days | 2. Refusal to sign the informed consent form |
| 3. High risk PE patients with absolute contraindications to ST or its failure (refractory circulatory collapse) not eligible for SE | 3. Presence of intracardiac thrombus  4. Diagnosed thrombophilia |
| 4. Intermediate-high risk PE patients with RV dysfunction confirmed by CTPA or transthoracic echocardiography and elevated troponin level with concomitant at least one of below criterium for minimum 24 hours:  a) SBP > 90 mmHg and ≤ 100 mmHg  b) HR ≥ 110/min,  c) SaO_2_ <90% during spontaneous breathing (atm) | 5. Severe thrombocytopenia (platelets count below 20 000 µL)  6. History of severe or chronic pulmonary hypertension  7. Serum creatinine level higher than 1.8 mg/dl  8. Known serious and uncontrolled sensitivity to radiographic agents |
| 5. Intermediate-high risk PE patients with RV dysfunction confirmed by CTPA or transthoracic echocardiography and elevated troponin level with sudden occurrence of one or more of the below listed factors:  a) SBP > 90 mmHg and ≤ 100 mmHg  b) HR ≥ 110/min,  c) SaO_2_ <90% during spontaneous breathing (atm) |  |

Table 2. Clinical presentation of study patients, indications to CDT and PE risk classification. CDT= catheter-directed thrombectomy, F=female, M=male, PE= pulmonary embolism

|  | Patient’s Initials, sex (M/F),  age (years) | Clinical presentation | Indication for CDT treatment | PE clinical risk category |
| --- | --- | --- | --- | --- |
| 1 | KL, M, 67 | Cardiogenic shock, cardiac arrest, intubated | Unsuccessful thrombolysis | High risk |
| 2 | EK, F, 44 | Sudden cardiac arrest, cardiopulmonary resuscitation>1 hour, intubated, ECMO delivery | Relative contraindications to thrombolysis | High risk |
| 3 | GB, M, 29 | Multiorgan trauma, intracranial hematoma, hypotension, intubated | Absolute contraindications  to thrombolysis | High risk |
| 4 | JŁ, F, 44 | Ovarian cancer after surgery and chemotherapy, no improvement on AC treatment, respiratory failure | Relative contraindications to thrombolysis | Intermediate-high risk |
| 5 | TW, F, 70 | Hemorrhagic stroke, respiratory failure | Absolute contraindications to thrombolysis | Intermediate-high risk |
| 6 | MK, F, 56 | 4 days after left lower limb surgery (femoral fracture), respiratory decompensation | Absolute contraindications to thrombolysis | Intermediate -high risk |
| 7 | AW, M, 59 | Colon cancer after surgery (7 days before), respiratory decompensation | Absolute contraindications to thrombolysis | Intermediate-high risk |
| 8 | IJ, F, 42 | 6 days after vascular surgery, presyncope | Absolute contraindications to thrombolysis | High risk |
| 9 | MK, M, 42 | Testicular tumor, respiratory failure | Relative contraindications to thrombolysis | Intermediate-high risk |
| 10 | AO, F, 47 | 2 days after abdominal surgery 2 (abdominoplasty, blepharoplasty), ischemic stroke, cardiac arrest, intubated | Absolute contraindication to thrombolysis | High risk |
| 11 | LP, M, 62 | Vascular surgery one day before (brachial artery thrombus), ischemic stroke, respiratory failure, hypotonia | Absolute contraindications to thrombolysis | Intermediate-high risk |
| 12 | SM, M, 62 | 4 days after surgery (lower limb ulceration), syncope, respiratory failure | Absolute contraindications to thrombolysis | Intermediate-high risk |
| 13 | DM, F, 52 | 7 days after major orthopedic surgery (left hip replacement), presyncope, respiratory failure | Absolute contraindications to thrombolysis | Intermediate-high risk |
| 14 | SJ, F, 67 | Post ischemic stroke, Parkinson disease, respiratory failure, desaturation, no improvement on UFH | Relative contraindications to thrombolysis | Intermediate-high risk |

Table 3. The clinical endpoints of the study.

CDT= catheter-directed mechanical aspiration thrombectomy, CTPA= computed tomography pulmonary angiography, HR= heart rate, PAP= pulmonary arterial pressure, PE=pulmonary embolism, RV= right ventricle, SaO_2_= arterial blood oxygen saturation, SBP= systolic blood pressure

| Endpoints of the study |
| --- |
| 1. Clinical improvement during CDT procedure (change in SBP, HR and SaO_2_). |
| 2. Improvement of PAPs during the CDT procedure. |
| 3. Reduction in vascular obstruction in the angiography measured at the end of CDT with Miller Index score. |
| 4. Change in RV strain in echocardiography measured 24 hours after the procedure. |
| 5. Death caused by PE (RV failure) during index hospitalization or follow-up period |
| 6. Death of any cause during index hospitalization or follow-up period |
| 7.CDT-related major adverse events (major bleeding, pulmonary vascular injury). |
